# Identification of Copy Number Variations in Familial Hemiplegic Migraine Genes in Suspected Hemiplegic Migraine Patients

**DOI:** 10.3390/biomedicines14050954

**Published:** 2026-04-22

**Authors:** Thais Zielke, Heidi G. Sutherland, Neven Maksemous, Robert A. Smith, Lyn R. Griffiths

**Affiliations:** Genomics Research Centre, Centre for Genomics and Personalised Health, School of Biomedical Sciences, Queensland University of Technology (QUT), Brisbane, QLD 4059, Australia; thaiszielke@hotmail.com (T.Z.); heidi.sutherland@qut.edu.au (H.G.S.); n.maksemous@qut.edu.au (N.M.); r157.smith@qut.edu.au (R.A.S.)

**Keywords:** familial hemiplegic migraine, hemiplegic migraine, copy number variations (CNVs), *CACNA1A*, *ATP1A2*, *SCN1A*, *PRRT2*

## Abstract

**Background**: Familial hemiplegic migraine (FHM) is a rare and severe form of migraine disorder featuring aura symptoms that include hemiplegia during attacks. While pathogenic missense variants in *CACNA1A*, *ATP1A2*, and *SCN1A* can cause FHM or its sporadic form, they explain less than 20% of suspected hemiplegic migraine cases, suggesting the involvement of other genes or genetic variations, potentially including copy number variations (CNVs). *PPRT2* gene variants including CNVs have also been implicated in hemiplegic migraine. **Methods**: Multiplex ligation-dependent probe amplification (MLPA) assays were used to investigate the presence of CNVs in the *CACNA1A*, *SCN1A*, *ATP1A2*, and *PRRT2* genes in a cohort of 170 unrelated probands suspected to have FHM who had tested negative for pathogenic missense or small indel variants within these genes. Potential CNVs were subsequently confirmed using quantitative PCR. **Results**: In 15 patients referred for FHM genetic testing, various CNVs in the target genes were detected by MLPA and subsequently validated by quantitative PCR. *CACNA1A* exon duplications were identified in six patients and deletions found in two. Two patients had *ATP1A2* exon deletions, while one had a duplication. For *SCN1A*, exon deletions were found in three patients and a duplication in one. *PRRT2* exon deletions were detected in five patients, with a single nucleotide polymorphism (SNP) array confirming a deletion spanning *PRRT2* and neighbouring loci including 26 genes in one of those. Three patients had CNVs in more than one FHM gene. **Conclusions**: Our study demonstrates the presence of CNVs in FHM genes in a subset of hemiplegic migraine cases (~9%), suggesting a likely role in the disorder and highlighting the need to explore structural variation in addition to the commonly interrogated genetic mutation points. These findings contribute to further understanding of genetic mechanisms that underlie hemiplegic migraine and may inform improved diagnostic and therapeutic strategies.

## 1. Introduction

Hemiplegic migraine is a rare subtype of migraine with aura in which individuals experience hemiplegia or unilateral weakness during a migraine attack, as well as other symptoms that commonly present in migraine [1]. When hemiplegic migraine is inherited—usually in an autosomal dominant manner—it is characterised as familial hemiplegic migraine (FHM); however, it may also occur in the absence of a family history (sporadic hemiplegic migraine—SHM) [2].

Three main causal genes have been identified for FHM—*CACNA1A*, *ATP1A2*, and *SCN1A*—which all encode ion channels or ion transport proteins [3]. Notably, pathogenic variants (e.g., missense, nonsense, splicing, small indels) in the FHM genes were detected in less than 20% of patients that were referred for testing to our diagnostic facility [4,5], and other researchers have similarly found a low diagnostic rate [6,7]. This suggests that other genes and/or other types of variants, such as copy number variants (CNVs) might be involved in disease aetiology [5,6,8].

Some studies have implicated other genes as potentially involved in hemiplegic migraine, including *PRRT2*, *PNKD*, *SLC1A3*, and *SLC4A4* [5,9]. In particular, *PRRT2*, encoding proline-rich transmembrane protein 2 (PRRT2), has been described as a fourth FHM gene, although clinical heterogeneity of individuals with *PRRT2* variants makes it difficult to assign causality [10,11,12,13]. As well as FHM, *PRRT2* has been implicated in other paroxysmal disorders, including paroxysmal kinesigenic dyskinesia, epilepsy, and episodic ataxia [12,14]. PRRT2 is a type-II transmembrane protein with important functions in various neurological processes during development, including neuronal migration, spinogenesis, formation and maintenance of synapses, and it moderates the presynaptic vesicle docking/priming process to regulate neurotransmitter release [15,16].

The genes associated with FHM play important roles in the excitatory and inhibitory ion balance within the central nervous system, and, critically, one of the main components thought to underlie migraine with aura is cortical spreading depression, an electrophysiological phenomenon characterised by a wave of excitation followed by depolarisation across the cortical neurons [17,18]. In a mouse model, *Prrt2* deficiency was found to reduce prolonged inactivation of voltage-gated sodium channels, leading to increased excitability and facilitated spreading depression generation in the cerebellar cortex [19].

Many hereditary disorders, including FHM, are caused by single nucleotide variants (SNVs) or small indels in the sequence of specific genes. However, copy number variants (CNVs) comprising duplications or deletions of large DNA segments are causal for some human Mendelian pathologies, as well as being implicated in complex genotypic and phenotypic traits [20,21,22]. The joint impact of chromosomal imbalances (25%) and CNVs (10%) accounts for as much as 35% of congenital disabilities [22,23]. Pathogenic CNVs have been found to be involved in a range of neurodevelopmental disorders such as intellectual disability, developmental delay, autism spectrum disorder, epilepsy, and other neurological phenotypes, as well as psychiatric disorders [22,24,25,26]. For movement disorders, muscle disorders and neuropathies, analysis of 4800 exomes found that CNVs contributed to ~7% of genetic diagnoses [27], and in some conditions CNVs are the most frequent cause of a genetic disease [28]. Only a handful of studies have screened for CNVs in hemiplegic migraine with *CACNA1A*, *ATP1A2*, and *PRRT2* CNVs identified in some patients [6,8,13,29,30].

Short-read sequencing technologies have been applied to CNV detection. However, the complex nature of variant content and, in particular, larger structural variants, makes CNV calling challenging and CNV callers consequently exhibit variable performance when handling short-read data [31]. Long-read sequencing technologies can provide better resolution for CNVs [31], but are associated with higher cost and may require specific sample preparation requirements. Multiplex ligation-dependent probe amplification (MLPA) is a non-sequencing-based multiplex PCR method of detecting CNVs, commonly used diagnostically due to accuracy, cost-effectiveness, and the availability of clinically relevant probe sets from MRC Holland [28].

In this study, we report a comprehensive CNV analysis targeting the key causative genes implicated in hemiplegic migraine (*CACNA1A*, *ATP1A2*, *SCN1A*, and *PRRT2*) within an Australian/New Zealand cohort of 170 patients referred for FHM gene testing who were negative for missense, splice site, or nonsense mutations within these genes. MLPA was used in this study as the primary methodology for detecting CNVs. Validated CNVs were detected in ~9% of patients, with some cases having more than one. Our results suggest that CNVs in FHM genes contribute to disease pathogenesis and that including their analysis in genetic testing may lead to improved diagnostic rates for the disorder.

## 2. Materials and Methods

This article presents findings derived from the PhD thesis of Thais Zielke, “Investigating the role of rare and common genetic variants in migraine subtypes” (2024), submitted to Queensland University of Technology [32].

### 2.1. Population Cohort

Patients from Australia and New Zealand exhibiting symptoms consistent with hemiplegic migraine, clinically diagnosed by a neurologist, provided blood samples for diagnostic testing of the main FHM genes (*CACNA1A*, *ATP1A2*, and *SCN1A*) at our Genomics Research Centre Diagnostic Testing Laboratory, Queensland University of Technology (QUT). Consent for genetic testing was obtained by their doctors and ethical approval for this study was provided by the QUT Human Research Ethics Committee (Project ID: 7416). Family history was reported for 25% of the cases, while 5% were reported as SHM, and family information was not provided for the remaining cases. Detailed clinical information aside from a suspected hemiplegic migraine diagnosis was not available for some patients if the clinician simply requested specific FHM gene testing.

### 2.2. Sequencing Analysis

For each of the patients referred for FHM gene testing comprehensive sequencing of the coding exons of *CACNA1A*, *ATP1A2* and *SCN1A* using a targeted 5-gene panel (which also includes *NOTCH3* and *KCNK18*) on the Ion Torrent next-generation sequencing (NGS) platform had previously been performed in our diagnostic facility [4]. Pathogenic variants within the three main FHM genes were identified in less than 25% of those patients. A total of 170 probands who were negative for pathogenic missense variants within *CACNA1A*, *ATP1A2* and *SCN1A*, and had sufficient DNA, were selected for this research.

### 2.3. MLPA Analysis

DNA samples from the probands were screened for potential CNVs using MLPA with three different available probe sets from MRC Holland that target the FHM-related genes of interest: P348-C1 (*CACNA1A*—13 exons; *ATP1A2*—21/23 exons; *PRRT2*—4 exons); P279 (*CACNA1A*—34 exons; *KCN1A*—2 exons); and P137 (*SCN1A*—26/29 exons). As noted by MRC-Holland these probe sets do not include *ATP1A2* exons 18 and 23 or *SCN1A* exons 27 to 29.

MLPA was performed according to the manufacturer’s instructions. The amplification products were then examined by capillary electrophoresis, on an Applied Biosystems^®^ 3500 genetic analyser (Thermo Fisher Scientific, Waltham, MA, USA). Relative quantification of the signal peaks from fragments of unique size generated by individual probes in the assay provided information about the template DNA copy number. Coffalyser.Net software v.240129.1959 (MRC Holland, Amsterdam, The Netherlands) was used to normalise the raw data and quantify the fragment peaks and determine the occurrence of CNVs.

### 2.4. Real Time Quantitative PCR

Suspected deletions and duplications identified through MLPA analysis were further validated via real time quantitative PCR (qPCR). Primer sequences to assay specific exons are shown in Supplementary Table S1. PCR cycle conditions were optimised for the primer sets and PCR efficiencies were calculated from standard curves and verified with linear regression to have R^2^ values above 0.99. Each reaction was performed with 15 ng of DNA, 5 pmol forward and reverse primers, GoTaq^®^ qPCR Master mix, CXR Reference Dye (Promega, Madison, WI, USA), and deionised water, totalling 10 µLs per well in 384-well optical plates. Thermal cycling was conducted on a QuantStudio Flex 7 (Thermo Fisher Scientific, Waltham, MA, USA) using a PCR cycling programme consisting of a pre-run activation step of 2 min at 95 °C, followed by 40 cycles of denaturation at 94 °C for 30 s, annealing at 60 °C for 30 s, and extension at 72 °C for 30 s, followed by a dissociation stage. All qPCR experiments were performed in triplicate.

Analysis of qPCR was performed using the 2^−ΔΔCT^ method [33,34]. Copy numbers for the exons with suspected CNVs in samples were determined by comparison with the known copy number of a control sample using *GAPDH* (Glyceraldehyde 3-phosphate dehydrogenase) as the reference gene.

### 2.5. SNP Array Analysis

For selected samples where large deletions were suspected, part of the validation method utilised SNP data obtained from the Illumina Infinium Global Screening Array chips with multi-disease content (GSAv3 + MD). The minimum sample input per reaction was 200 ng and all samples were normalised prior to submission, with the array hybridization and visualisation performed using manufacturer protocols by the Australian Translational Genomics Centre, QUT. Analysis was performed using CNVPartition, a plug-in for Genome Studio (Illumina, Inc., San Diego, CA, USA), in which calculations of CNVs were made through Log R Ratio and B Allele frequency.

## 3. Results

### 3.1. MLPA and qPCR Results

**Figure 1:**
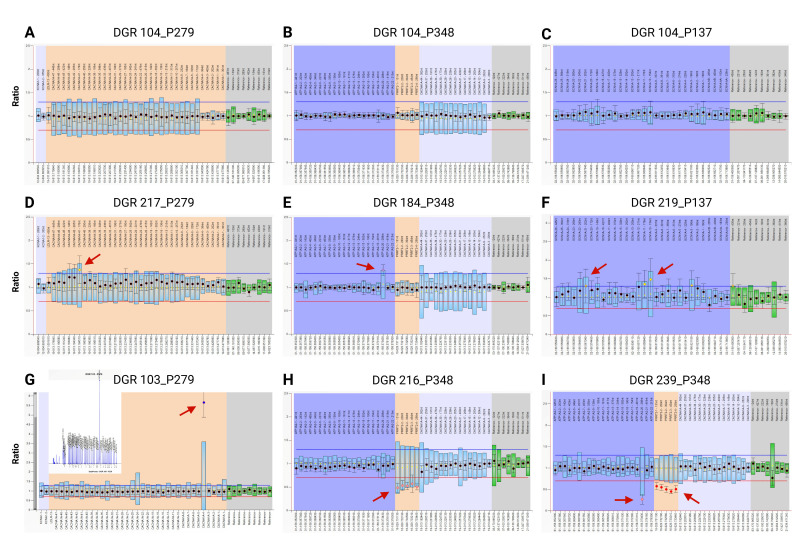
MLPA results showing CNVs in FHM genes in hemiplegic migraine patients. Examples of Coffalyser ratio plots of MLPA analysis with MRC Holland P279 (*CACNA1A* exons), P348 (*ATP1A2*, *PRRT2*, and *CACNA1A* exons) and P137 (*SCN1A* exons) probe sets. Colour of dots indicates significance information: black—no significant difference; yellow—ambiguous; purple—increased by 1–2 Standard Deviations (SDs); blue—increased by ≥2 SDs; orange—decreased by 1–2 SDs; red—decreased by ≥2 SDs. (**A**–**C**) Analysis of DGR 104 showing no CNVs across the four genes. Red arrows indicate CNVs detected in samples: (**D**) duplication of *CANCA1A* exon 41 in DGR 217; (**E**) duplication of *ATP1A2* exon 20 in DGR 184; (**F**) duplications of *SCN1A* exons 10, 11, and 21 in DGR 219; (**G**) five-fold amplification of *CACNA1A* exon 4 in DGR 103 with inset showing electropherogram from this sample; (**H**) deletion of all exons (1–4) of *PRRT2* in DGR 216; (**I**) deletion of all exons (1–4) of *PRRT2* as well as deletion of *ATP1A2* exon 20 in DGR 239. Figure created in BioRender. Sutherland, H. (2026) https://BioRender.com/8pk5r2x (accessed on 24 December 2025).

A total of 170 DNA samples were available from patients diagnosed with symptoms of hemiplegic migraine and referred for FHM gene testing, but negative for pathogenic mutations via NGS panel [4]. These were screened for CNVs in *CACNA1A*, *ATP1A2*, *SCN1A*, and *PRRT2* using MLPA assays with the P137, P279 and P348 probe sets from MRC Holland. Examples of the ratio plots obtained from the electropherograms for selected samples using Coffalyser.Net software are shown in Figure 1A–I). For the majority of DNA samples CNVs were not detected with the FHM gene MLPA probe sets (e.g., DGR 104, Figure 1A–C). However, following initial evaluation of MLPA results, suspected CNVs in 31 patient DNAs were identified as annotated in Supplementary Table S2.

As MPLA assays can be sensitive to the quality or salt concentrations of DNA, qPCR was subsequently used to further validate all the suspected CNVs. Not all CNVs identified using the MLPA were confirmed through qPCR, and some of the results obtained from both MLPA and qPCR analyses were inconclusive (Supplementary Table S2). Nevertheless, among the hemiplegic migraine samples analysed, a total of 15 cases exhibited confirmed CNVs involving the four targeted genes, as seen in Table 1. Interestingly, within this subset, three samples (DGR 112, DGR 146, and DGR 239) displayed multiple CNVs affecting more than one of the investigated genes.

Six hemiplegic patients exhibited amplifications in *CACNA1A*, while two had deletions within this gene. Notably, four individuals had duplications of exon 41—DGR 92, DGR 114, DGR 217 (Figure 1D), and DGR 112 (which also had a *SCN1A* exon 24 deletion). A five-fold amplification of *CACNA1A* exon 4 was observed in DGR 103 by MLPA (Figure 1G), which was confirmed by qPCR (Supplementary Table S2). With respect to *CACNA1A* deletions, a heterozygous exon 8 deletion was observed in DGR 204, and DGR 146 had a complex array of CNVs which included deletion of *CACNA1A* exon 1, as well as exons 41 to 44.

A number of confirmed CNVs featuring exon 20 of *ATP1A2* were detected in the hemiplegic migraine cohort. One sample (DGR 184) exhibited a heterozygous duplication of exon 20 (Figure 1E), while DGR 227 and DGR 239 had deletions of *ATP1A2* exon 20. Notably, the latter patient also had a large deletion which included all four *PRRT2* exons (Figure 1I and see below).

*SCN1A* variants can cause FHM or epilepsy (usually Dravet Syndrome), depending on their effect on channel function [35]. Two probands exhibited heterozygous deletions in *SCN1A*: DGR 123 had a heterozygous deletion of exons 18 to 21, while DGR 112 had a deletion of *SCN1A* exon 24 in addition to a *CACNA1A* exon 41 duplication. Heterozygous duplications of *SCN1A* exons 10, 11 and 21 were detected in DGR 219 (Figure 1F).

Heterozygous deletions of *PRRT2*, the most recently implicated FHM gene, were identified in five hemiplegic migraine patients. All exons of the gene were deleted in DGR 146, DGR 216 (Figure 1H), and DGR 239 (Figure 1I), while deletions encompassing exon 1 were confirmed in two other two probands (DGR 145 and DGR 189).

### 3.2. SNP Array Results

SNP arrays were performed for all samples and the FHM genes were examined for evidence of the CNVs identified via MLPA and qPCR. Only one deletion encompassing the entire *PRRT2* gene in DGR 239 was validated through this method. In fact, the SNP array for DGR 239 showed that this deletion affected a large genomic region spanning approximately 610.5 kilobase pairs (kbp) at chromosome 16p11.2. As listed in Table 2, the region includes approximately 26 genes including *PRRT2* and is a known recurrent microdeletion (16p11.2) [36]. The other validated CNVs in Table 1 were not detectable in the microarray data, suggesting that SNP array analysis is not particularly useful for detection of smaller CNVs.

## 4. Discussion

In this study, we conducted a comprehensive analysis of CNVs in the primary causal genes associated with hemiplegic migraine—*CACNA1A*, *SCN1A*, *ATP1A2*, and *PRRT2*—in a cohort comprising 170 patients clinically diagnosed with the disorder, but negative for pathogenic missense variants or small indels within these genes. MLPA analysis revealed the presence of both deletions and duplications within the FHM genes which were confirmed by qPCR in 15 probands as summarised in Table 1 and Supplementary Table S2. Notably, CNVs involving more than one FHM gene were found in three patients, which may interact through shared pathways.

*CACNA1A* encodes Voltage-Gated Calcium Channel Subunit Alpha1 A, the pore-forming subunit of Cav2.1, a major calcium channel in the brain. Duplications in *CACNA1A* were observed in six samples, while two samples exhibited deletions within the same gene. One notable finding was a five-fold amplification in exon 4 of *CACNA1A* in DGR 103, which would be likely to have functional implications in hemiplegic migraine pathogenesis for this patient. Interestingly, most of the observed duplications in *CACNA1A* included exon 41 (five out of six probands), suggesting that this genomic region may serve as a mutational hotspot for CNVs within the *CACNA1A* gene. Grieco et al. (2018), investigating CNVs in *CACNA1A* in hemiplegic migraine and episodic ataxia patients, also found deletions that included exon 41 in some hemiplegic migraine patients [6], further supporting the premise that this region is prone to genetic alteration and may play a significant role in hemiplegic migraine pathophysiology. A high frequency of deletions of *CACNA1A* exon 47 (with or without other exons) was also found in their cohort [6]. Copy number changes in *CACNA1A* are frequently associated with episodic ataxia; breakpoints often located in *Alu* repeats which may be involved in generating amplifications and deletions [29,37,38,39]. There is considerable overlap in symptoms between hemiplegic migraine patients and episodic ataxia patients, and the conditions may co-occur. Further investigation of copy number and other potential modifying susceptibility variants (including other CNVs) may lead to a better understanding of their impact on patients and more accurate diagnosis of these disorders.

One patient (DGR 184) exhibited an amplification specifically within exon 20 of *ATP1A2*. *ATP1A2* encodes the α2 catalytic subunit of the ATPase Na^+^/K^+^ pump, which plays an important role in establishing and maintaining the electrochemical gradients which regulate neurotransmitter uptake and muscle contraction [40]. Gagliardi et al. (2017) suggest that *ATP1A2* duplication might lead to elevated levels of extracellular potassium and intracellular sodium, resulting in increased intracellular calcium levels through the Na^+^/Ca^2+^ exchanger [8]. This would lead to increased synaptic glutamate, which can trigger cortical spreading depression, thought to underlie the aura that features in some migraine subtypes. Heterozygous deletions of exon 20 were found in DGR 227 and DGR 239. These findings highlight the potential involvement of different types of CNVs in this gene. Epilepsy may arise through *ATP1A2* loss of function potentially including via deletions [41]. Although typically distinct, some studies have documented cases where FHM2 and epilepsy coexist within the same individual [42,43], suggesting similar pathogenic mechanisms affecting ion channels and pump function, although with other factors that may modify expression to manifest the different conditions [44]. Interestingly, DGR 239 was another example of a patient with a complex landscape of FHM gene-related CNVs, as additional to the *ATP1A2* exon 20 deletion they had the 16p11.2 microdeletion which includes *PRRT2*.

Investigation of *PRRT2* identified five samples with heterozygous deletions, further supporting a role for this gene in hemiplegic migraine. Riant et al. (2022) detected eight of 860 hemiplegic migraine probands with deletions of the whole *PRRT2* gene [13]. Four of these had a deletion of ~546 kb at the 16p11.2 locus, similar to that the recurrent CNV of 16p11.2 deletion syndrome [36], and the deletion found in DGR 239 in this study. Among our cohort, another two individuals had heterozygous deletions spanning all four exons of *PRRT2*, but not large enough to be detected by microarray, and two other probands exhibited partial *PRRT2* deletions. Chromosome 16 harbours highly homologous low-copy repeats, which facilitate genetic rearrangements through nonallelic homologous recombination events during meiosis [45]. Our findings underscore the presence of various types of deletions in this gene and the significance of *PRRT2* in hemiplegic migraine pathogenesis.

*PRRT2* is known to exert crucial roles in brain development, neurogenesis, synapse formation, and calcium-dependent neurotransmitter release [46]. *PRRT2* deletions, particularly in the context of the larger 16p11.2 deletion, may also be associated with developmental delay phenotypes, as observed in the study conducted by Riant et al. (2022) [13]. Developmental delay was also a clinical feature of patient DGR 216 in this study, who had a heterozygous deletion of all exons of *PRRT2* (and possibly other genes not detected by the MLPA assay). The clinical spectrum of neurocognitive phenotypes associated with 16p11.2 deletions is variable. Patients may present with intellectual disability, morbid obesity, large head circumference, epilepsy and other symptoms, each displaying varying degrees of penetrance [45]. This clinical heterogeneity and incomplete penetrance poses challenges for accurate clinical interpretation and diagnosis of 16p11.2 deletion patients as genotype–phenotype correlations are complex [45]. Multiple genes within the region may influence phenotypic expression, either singly, or through interaction with other genetic factors; detailed molecular investigations are needed to understand the underlying mechanisms of the diverse clinical presentations observed in patients with 16p11.2 deletions [47].

Analysis of *SCN1A*, which encodes the pore-forming subunit of the Nav1.1 sodium channel, revealed heterozygous deletions in two hemiplegic patients (DGR 112 and DGR 123) and amplifications across three distinct exons in another (DGR 219). Previous studies have shown that Nav1.1 channels regulate the excitability of neurons via mediating the depolarizing phase of action potentials in excitable membranes, and that deletions in *SCN1A* can cause Dravet syndrome and epilepsy [48,49]. Deletions in *SCN1A* have not been previously reported in hemiplegic migraine; rather, gain-of-function *SCN1A* variants are typically linked to FHM3 [50]. Therefore, together with studies identifying *SCN1A* point mutations in FHM, our findings suggest that CNVs in *SCN1A* may also be causal in some patients.

Limitations in our investigation included the availability of comprehensive clinical data for all enrolled patients which may impede clear understanding of genotype–phenotype relationships, and that the cohort comprised probands referred for diagnostic testing and other family members were unavailable for segregation analysis. Nevertheless, comparing available clinical information with patients with classical pathogenic variants in FHM genes showed a similar range of phenotypes as those with CNVs, such as hemiplegic migraine episodes, family history of migraine/FHM, and some cases with onset of symptoms from an early age and developmental delay [4]. On the other hand, in patients in which no pathogenic variant has yet been identified, additional varied symptoms were reported in some patients, such as vertigo and brain stem aura features, stroke-like episodes, pyramidal signs, and late onset of symptoms. This suggests that this group may represent a more genetically and phenotypically diverse cohort, and possible confounding factors could include non-motor complex auras [51,52], migraine with unilateral motor symptoms (MUMS) [53], or cerebral small vessel disease [54] (although with respect to the latter, patients had been excluded for pathogenic *NOTCH3* variants).

Validating some of the CNVs detected by MLPA via qPCR was challenging, as the estimated copy number was inconclusive in some cases and they could not be confirmed as true CNVs. Further, SNP array analysis was only able to confirm one large deletion in a single patient, underlining that SNP arrays are more useful for larger CNV detection. We also acknowledge that due to cost constraints we did not perform MLPA assays on non-migraine controls in this study to determine whether any of these specific CNVs are present in the general population.

To extend understanding of such CNVs in FHM, other approaches may be undertaken in the future. For instance, long-read sequencing techniques could offer more precise identification of the breakpoints, boundaries, and arrangements of these structural genomic alterations. Additionally, gene expression studies utilising RNA sequencing would be beneficial to understand the impact of the CNVs on transcript structure and gene expression at each loci [55,56]. Future studies incorporating functional analyses, using derived patient-specific cell lines or CRISPR-Cas9-based gene-editing techniques to introduce CNVs into cell lines could contribute to elucidating the consequences of these specific CNVs when compared with controls [57,58,59,60].

## 5. Conclusions

We report the presence of CNVs in known FHM causal genes in a subset of hemiplegic migraine cases in the absence of pathogenic SNV or small indels in these genes, expanding our understanding of the genetic factors that underly this condition. The identification of CNVs in *CACNA1A*, *ATP1A2*, *SCN1A*, and *PRRT2*, as well as the co-occurrence of CNVs in multiple genes, underscores the complexity of the genetic landscape in hemiplegic migraine. With classical pathogenic variants (missense, nonsense, splicing, small indels) identified in only ~20% of referred patients with symptoms of hemiplegic migraine, finding a molecular diagnosis in an additional ~9% of individuals represents a substantial uplift in diagnostic rate. Furthermore, this knowledge may be translated into clinical practise by treating the patient with drugs that target, or are effective for, the affected ion channel or ATPase, similar to what occurs for cases with classical pathogenic variants [61,62]. Thus, this comprehensive analysis of CNVs, particularly the confirmation of *PRRT2* deletions and duplications found in exon 41 of *CACNA1A*, provides valuable insights and a broader understanding of the underlying molecular basis of hemiplegic migraine and may inform improved diagnostic strategies and targeted therapeutic approaches for affected individuals.

## Figures and Tables

**Table 1:** Summary of confirmed CNVs in FHM genes identified in hemiplegic patients.

Sample ID	Gene	Exons	CNV	Clinical Features
DGR 92	*CACNA1A*	41	Duplication	FHM gene testing. No additional clinical features provided.
DGR 103	*CACNA1A*	4	5-fold amplification	Hemiplegic migraine in the past. Prolonged episode of right cortical signs lasting for days.
DGR 112	*CACNA1A*	41	Duplication	FHM gene testing. No additional clinical features provided.
	*SCN1A*	24	Deletion	
DGR 114	*CACNA1A*	20, 41	Duplication	Migraine, seizures post-ictally, Todd’s palsy.
DGR 123	*SCN1A*	18–21	Deletion	Migraine, coma, acute respiratory failure, family history of FHM.
DGR 145	*PRRT2*	1	Deletion	FHM gene testing. No additional clinical features provided.
DGR 146	*CACNA1A*	1, 41–44	Deletion	FHM gene testing. No additional clinical features provided.
	*CACNA1A*	9	Duplication	
	*PRRT2*	1–4	Deletion	
DGR 184	*ATP1A2*	20	Duplication	FHM gene testing. No additional clinical features provided.
DGR 189	*PRRT2*	1	Deletion	Severe headaches with aura, paralysis of right side of the body. Strong family history of FHM.
DGR 204	*CACNA1A*	8	Deletion	Migraine-like seizures, progressive occipital lobe injury, numbness, lateralised weakness, aphasia, headache and hemideficit lasting days.
DGR 216	*PRRT2*	1–4	Deletion	Seizures, hemiplegia, developmental delay. Sibling has similar symptoms and mother has headaches and facial weakness.
DGR 217	*CACNA1A*	41	Duplication	Hemiplegic migraine on right side.
DGR 219	*SCN1A*	10, 11, 21	Duplication	Family history of FHM.
DGR 227	*ATP1A2*	20	Deletion	Seizures, right facial drop, persistent headaches.
DGR 239	*ATP1A2*	20	Deletion	Headache with right-sided weakness (hemiplegia), mother had similar episode.
	*PRRT2*	1–4	Deletion	

**Table 2:** CNV findings in SNP array for DGR 239 confirming deletion of genes including *PRRT2*.

Sample ID	Chr	Start	End	Size in bp	Gene IDs
DGR 239	16	29595483	30206047	610564	*BOLA2B*, *SLC7A5P1*, *SPN*, *QPRT*, ***PRRT2***, *KIF22*, *MAZ*, *PAGR1*, *MVP*, *CDIPT*, *SEZ6L2*, *ASPHD1*, *KCTD13*, *TMEM219*, *TAOK2*, *HIRIP3*, *INO80E*, *DOC2A*, *TLCD3B*, *ALDOA*, *C16orf92*, *CTD-2515O10.6*, *PPP4C*, *TBX6*, *YPEL3*, *CORO1A*, *GPD3*, *MAPK3*, *RP11-455F5.3*

The *PRRT2* gene is bolded to highlight it and its position with respect to other gene encompassed by the 610.5 kbp chromosome 16p11.2 deletion.

## Data Availability

All data supporting the findings of this study are available within the paper and Supplementary Tables.

## Supplementary Materials

The following supporting information can be downloaded at: https://www.mdpi.com/article/10.3390/biomedicines14050954/s1, Table S1: Sequences of qPCR primers and amplicons for CNV validations, Table S2: Summary of qPCR analysis.

## Abbreviations

The following abbreviations are used in this manuscript:

CNV	Copy number variation
FHM	Familial hemiplegic migraine
MLPA	Multiplex ligation-dependent probe amplification
NGS	Next generation sequencing
PCR	Polymerase chain reaction
qPCR	Quantitative PCR
QUT	Queensland University of Technology
SHM	Sporadic hemiplegic migraine
SNP	Single nucleotide polymorphism
SNV	Single nucleotide variants
