# Supplementary material for: Identification of Copy Number Variations in Familial Hemiplegic Migraine Genes in Suspected Hemiplegic Migraine Patients
Source: Biomedicines. 2026 Apr 22;14(5):954. doi: 10.3390/biomedicines14050954 (PMC13203776; doi:10.3390/biomedicines14050954)
Supplement: Supplementary file 1 [file biomedicines-14-00954-s001.zip › Supplementary Table S1 qPCR primers.pdf]

Supplementary Table S1. Sequences of qPCR primers and amplicons for CNV validations

**Description**	**Forward**	**Reverse**	**Size**
***PRRT2*-Exon 1**	CCTCCCTAGCTGACTTGCTC	TCGGTCGGTCCACTCCTA	135bp
***PRRT2*-Exon 2**	GGCTCCAGAAACCACAGAGA	GTGGCCTCTTTGCTCACTTC	156bp
***PRRT2*-Exon 3**	AGCCAAGCTCTTAAGCATCG	AGGGGCAGGGGTTAGTAAAG	151bp
***PRRT2*-Exon 4**	CGTCTGTCCTTCCCTCTCCT	CAGGCTCCCTTGGTCCTTA	162bp
***ATP1A2*-Exon 3**	TAACTCACCCTCCCTTCCCC	TTTAAGGCTCCTGCCACCTT	159bp
***ATP1A2*-Exon 7**	CTGTGTGCATACAAGTGGCTC	GAAACAGATATTGCGGGTCTCC	153bp
***ATP1A2*- Exon 9**	GTCCACCATCTGCTCGGACAA	AATCCTATCCACCCCCTCTGGA	147bp
***ATP1A2*- Exon 20**	ACCCTTTCCTCCGACACTCT	TGCAGATGATGAGGTCAGCC	142bp
***CACNA1A*- Exon 1**	CCATTCGGTGATCTTTTTGGCG	AAGCAGTCAATGGCGCAGAG	147bp
***CACNA1A*- Exon 4**	TCAGGGTCGGAAACTCACG	TTCTTTCAGCATCTTGGCGAC	123bp
***CACNA1A*- Exon 8**	GCATGACTCTCTTTGTACTCCGT	GCGGCAACAACAGATTGAACG	149bp
***CACNA1A*- Exon 9**	GAGGTGGGTTTAGAGCAGTTACCA	AGGAGCTTAACTCTGTGCTCTTCC	110bp
***CACNA1A*- Exon 17**	CAGGTGGTAACTTTGCCAGAGAAA	TTTGCAGACACCCTCCTGAATG	119bp
***CACNA1A*- Exon 20**	ACGATAAGGCTATTCTCGGGGG	CCAAGATGGGAAACAGCACCG	154bp
***CACNA1A*- Exon 24**	GAACCCTTGCGAGGAGACTTA	TCCCCCACAGATGATTGACC	139bp
***CACNA1A*- Exon 41**	AGCTTCTTGGCCTTGCTCTG	TGAGCTCACCGTGTGTGTG	105bp
***CACNA1A*- Exon 44**	GTGAAAGCCCTCACCTGGTTCT	GATGGCTACTCCGACAGCGA	101bp
***SCN1A*_Exon_5**	GTCATTACATTTGCGTACGTCACAGAG	TCAGGCCTGGAATGACTGAAATCG	112bp
***SCN1A*_Exon_9**	TTTGATCCTGGCTGTGGTGG	AGTTGCCGTTGCTGCCT	148bp
***SCN1A*_Exon_10**	GACAGCTCATCTGAAGCCTCTA	TGATGCTGTCCTCAGATTCAGATT	148bp
***SCN1A*_Exon_11**	GAGCACAGCACCTTTGAGGA	CATTCGCTGGAAACACTGCC	136bp
***SCN1A*_Exon_18**	GGTGGAACCTGAGAAGGACG	TCAAATGCCAGAGCACCACT	103bp
***SCN1A*_Exon_21**	TCCATCATGAATGTGCTTCTGGT	TCACGTCTTCGATGTCAAACCT	136bp
***SCN1A*_Exon_25**	GGTCTTTGACTTCGTAACCAGA	TGAACACCAGATTGATGCGTG	143bp
***SCN1A*_Exon_26**	AGAGCGATTCATGGCTTCCA	GGAAGCTTGTTTTACAGTTCGCT	148bp
GAPDH	GGAAACCAGATCTCCCACCG	GTGTGTGGGGAGAAGGGATG	110bp
