# Supplementary material for: Identification of Copy Number Variations in Familial Hemiplegic Migraine Genes in Suspected Hemiplegic Migraine Patients
Source: Biomedicines. 2026 Apr 22;14(5):954. doi: 10.3390/biomedicines14050954 (PMC13203776; doi:10.3390/biomedicines14050954)
Supplement: Supplementary file 1 [file biomedicines-14-00954-s001.zip › Supplementary Table S2 Summary of qPCR results.pdf]

Supplementary Table S2. Summary of qPCR analysis

DGR	ID	Gene	Exon	MLPA analysis	$2^{-\Delta\Delta Ct}$	qPCR analysis	Validated
**DGR**	63	*CACNA1A*	24	Duplication	1.86	Normal copy number	No
**DGR**	92	*CACNA1A*	41	Duplication	3.9	Homozygous duplication or heterozygous amplification	Yes
**DGR**	103	*CACNA1A*	4	5-fold amplification	10.45	5-fold amplification	Yes
**DGR**	112	*ATP1A2*	7	Duplication	1.95	Normal copy number	No
**DGR**	112	*CACNA1A*	8	Duplication	0.9	Suggestive of deletion	No
**DGR**	112	*CACNA1A*	41	Duplication	3.01	Heterozygous duplication	Yes
**DGR**	112	*SCN1A*	24	Deletion	1.1	Heterozygous deletion	Yes
**DGR**	113	*SCN1A*	5	Duplication	0.16	Heterozygous deletion	No
**DGR**	114	*CACNA1A*	1	Duplication	1.29	Inconclusive	No
**DGR**	114	*CACNA1A*	4	Duplication	1.1	Suggestive of deletion	No
**DGR**	114	*CACNA1A*	8	Duplication	1.25	Suggestive of deletion	No
**DGR**	114	*CACNA1A*	20	Duplication	2.7	Heterozygous duplication	Yes
**DGR**	114	*CACNA1A*	24	Duplication	2.14	Normal copy number	No
**DGR**	114	*CACNA1A*	41	Duplication	3.67	Heterozygous duplication	Yes
**DGR**	123	*SCN1A*	18	Deletion	0.91	Heterozygous deletion	Yes
**DGR**	123	*SCN1A*	21	Deletion	1.05	Heterozygous deletion	Yes
**DGR**	141	*ATP1A2*	20	Deletion	2.04	Normal copy number	No
**DGR**	142	*ATP1A2*	20	Deletion	1.55	Inconclusive	No
**DGR**	145	*CACNA1A*	1	Duplication	1.38	Inconclusive	No
**DGR**	145	*CACNA1A*	17	Deletion	1.7	Inconclusive	No
**DGR**	145	*CACNA1A*	24	Duplication	2.5	Inconclusive	No
**DGR**	145	*CACNA1A*	41	Deletion	5.13	Amplification	No
**DGR**	145	*CACNA1A*	44	Deletion	1.7	Inconclusive	No
**DGR**	145	*PRRT2*	1	Deletion	1.29	Heterozygous deletion	Yes
**DGR**	145	*PRRT2*	2	Deletion	1.57	Inconclusive	No
**DGR**	145	*PRRT2*	3	Deletion	1.57	Inconclusive	No
**DGR**	145	*PRRT2*	4	Deletion	2.4	Normal copy number	No
**DGR**	146	*ATP1A2*	3	Duplication	2.16	Normal copy number	No
**DGR**	146	*ATP1A2*	9	Duplication	1.88	Normal copy number	No
**DGR**	146	*CACNA1A*	1	Deletion	1.1	Heterozygous deletion	Yes
**DGR**	146	*CACNA1A*	9	Duplication	2.93	Heterozygous duplication	Yes
**DGR**	146	*CACNA1A*	17	Deletion	1.59	Inconclusive	No
**DGR**	146	*CACNA1A*	20	Deletion	1.6	Inconclusive	No
**DGR**	146	*CACNA1A*	41	Deletion	0.72	Heterozygous deletion	Yes
**DGR**	146	*CACNA1A*	44	Deletion	0.7	Heterozygous deletion	Yes
**DGR**	146	*PRRT2*	1	Deletion	0.87	Heterozygous deletion	Yes
**DGR**	146	*PRRT2*	2	Deletion	1.27	Heterozygous deletion	Yes
**DGR**	146	*PRRT2*	3	Deletion	1.24	Heterozygous deletion	Yes

**DGR**	146	*PRRT2*	4	Deletion	1.3	Heterozygous deletion	Yes
**DGR**	146	*SCN1A*	5	Duplication	2.32	Normal copy number	No
**DGR**	146	*SCN1A*	18	Deletion	2.1	Normal copy number	No
**DGR**	146	*SCN1A*	25	Duplication	2.26	Normal copy number	No
**DGR**	154	*ATP1A2*	20	Deletion	2.63	Inconclusive	No
**DGR**	184	*ATP1A2*	20	Duplication	3.04	Heterozygous duplication	Yes
**DGR**	189	*PRRT2*	1	Deletion	1.19	Heterozygous deletion	Yes
**DGR**	189	*PRRT2*	2	Deletion	1.97	Normal copy number	No
**DGR**	189	*SCN1A*	5	Duplication	2.17	Normal copy number	No
**DGR**	189	*SCN1A*	10	Duplication	1.93	Normal copy number	No
**DGR**	189	*SCN1A*	11	Duplication	2.09	Normal copy number	No
**DGR**	189	*SCN1A*	21	Duplication	2.08	Normal copy number	No
**DGR**	194	*CACNA1A*	8	Deletion	4.05	Suggestive of duplication	No
**DGR**	197	*ATP1A2*	9	Deletion	2.12	Normal copy number	No
**DGR**	201	*CACNA1A*	8	Deletion	2.1	Normal copy number	No
**DGR**	204	*CACNA1A*	8	Deletion	1	Heterozygous deletion	Yes
**DGR**	205	*CACNA1A*	8	Deletion	1.7	Inconclusive	No
**DGR**	206	*ATP1A2*	9	Deletion	1.88	Normal copy number	No
**DGR**	207	*CACNA1A*	8	Deletion	1.8	Normal copy number	No
**DGR**	212	*ATP1A2*	9	Deletion	1.62	Inconclusive	No
**DGR**	216	*PRRT2*	1	Deletion	1.04	Heterozygous deletion	Yes
**DGR**	216	*PRRT2*	2	Deletion	1.27	Heterozygous deletion	Yes
**DGR**	216	*PRRT2*	3	Deletion	1.3	Heterozygous deletion	Yes
**DGR**	216	*PRRT2*	4	Deletion	1.15	Heterozygous deletion	Yes
**DGR**	217	*CACNA1A*	41	Duplication	5.87	3-fold amplification?	Yes
**DGR**	219	*ATP1A2*	9	Deletion	2.8	Heterozygous duplication	No
**DGR**	219	*SCN1A*	10	Duplication	2.95	Heterozygous duplication	Yes
**DGR**	219	*SCN1A*	11	Duplication	2.94	Heterozygous duplication	Yes
**DGR**	219	*SCN1A*	21	Duplication	2.75	Heterozygous duplication	Yes
**DGR**	220	*ATP1A2*	9	Deletion	1.85	Normal copy number	No
**DGR**	220	*SCN1A*	21	Duplication	1.6	Inconclusive	No
**DGR**	220	*SCN1A*	25	Duplication	1.93	Normal copy number	No
**DGR**	223	*SCN1A*	10	Duplication	2.14	Normal copy number	No
**DGR**	223	*SCN1A*	21	Duplication	2.07	Normal copy number	No
**DGR**	227	*ATP1A2*	20	Duplication	3.07	Heterozygous duplication	Yes
**DGR**	239	*ATP1A2*	20	Deletion	1.11	Heterozygous deletion	Yes
**DGR**	239	*PRRT2*	1	Deletion	1.07	Heterozygous deletion	Yes
**DGR**	239	*PRRT2*	2	Deletion	1.19	Heterozygous deletion	Yes
**DGR**	239	*PRRT2*	3	Deletion	1.08	Heterozygous deletion	Yes
**DGR**	239	*PRRT2*	4	Deletion	1.08	Heterozygous deletion	Yes
**DGR**	242	*ATP1A2*	20	Deletion	2.27	Normal copy number	No
**DGR**	255	*ATP1A2*	20	Duplication	2.45	Inconclusive	No

Note: The $2^{-\Delta\Delta Ct}$ is based on a control proband with normal copy number of 2 (1 copy for each allele), a heterozygous deletion will be close to 1, homozygous deletion would be 0, duplications/amplifications are close to 3 or more.
